# β-Hydroxy carbocation intermediates in solvolyses of di- and tetra-hydronaphthalene substrates

**DOI:** 10.3762/bjoc.6.118

**Published:** 2010-11-03

**Authors:** Jaya S Kudavalli, Rory A More O'Ferrall

**Affiliations:** 1 School of Chemistry and Chemical Biology, University College Dublin, Belfield, Dublin 4, Ireland

**Keywords:** aromaticity, β-hydroxycarbocations, hyperconjugation, solvolysis

## Abstract

Solvolysis of trichloroacetate esters of 2-methoxy-1,2-dihydro-1-naphthols shows a remarkably large difference in rates between the *cis* and *trans* isomers, *k_cis_*/*k_trans_* = 1800 in aqueous acetonitrile. This mirrors the behaviour of the acid-catalysed dehydration of *cis*- and *trans*-naphthalene-1,2-dihydrodiols to form 2-naphthol, for which *k_cis_*/*k_trans_* = 440, but contrasts with that for solvolysis of tetrahydronaphthalene substrates, 1-chloro-2-hydroxy-1,2,3,4-tetrahydronaphthalenes, for which *k_cis_*/*k_trans_* = 0.5. Evidence is presented showing that the *trans* isomer of the dihydro substrates reacts unusually slowly rather than the *cis* isomer unusually rapidly. Comparison of rates of solvolysis of 1-chloro-1,2,3,4-tetrahydronaphthalene and the corresponding (*cis*) substrate with a 2-hydroxy group indicates that a β-OH slows the reaction by nearly 2000-fold, which represents a typical inductive effect characteristic also of *cis*-dihydrodiol substrates. The slow reaction of the *trans*-dihydrodiol substrate is consistent with initial formation of a β-hydroxynaphthalenium carbocation with a conformation in which a C–OH occupies an axial position β to the carbocation centre preventing stabilisation of the carbocation by C–H hyperconjugation, which would occur in the conformation initially formed from the *cis* isomer. It is suggested that C–H hyperconjugation is particularly pronounced for a β-hydroxynaphthalenium ion intermediate because the stability of its no-bond resonance structure reflects the presence of an aromatic naphthol structure.

## Introduction

**Scheme 1:**
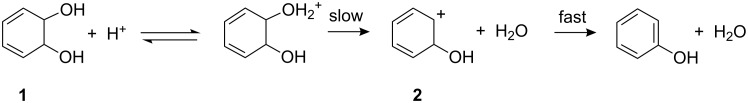
Mechanism of dehydration of benzene-1,2-dihydrodiol.

*Cis*-arenedihydrodiols are products of fermentation of aromatic molecules by mutant strains of soil bacteria containing dioxygenase enzymes such as *Pseudomonas putida* UV4 [1]. A characteristic reaction they undergo is acid-catalysed dehydration to form phenols [2], as illustrated for benzene-1,2-dihydrodiol in Scheme 1.

**Figure 1:**
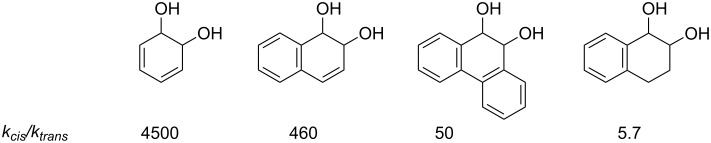
Reactivity ratios for acid-catalyzed reaction of arene dihydrodiols.

A surprising finding is that the reactivity of *cis*-dihydrodiols is much greater than that of the synthetically accessible *trans* isomers, e.g., *k_cis_*/*k_trans_* = 4500 for *cis*- and *trans*-benzene dihydrodiols (**1**), despite the expectation that the two reactions proceed through a common β-hydroxycarbocation intermediate (**2**) [3]. This contrasts with reactions of *cis*- and *trans*-dihydrodiols of non-aromatic double bonds for which only a small advantage for the *cis*-diol is observed. Indeed, the *cis*/*trans* rate ratio decreases regularly as the aromaticity of the double bond decreases, as shown for the dihydrodiols of benzene, naphthalene (1,2), phenanthrene (9,10) and 3,4-dihydronaphthalene in Figure 1 [3].

**Figure 2:**
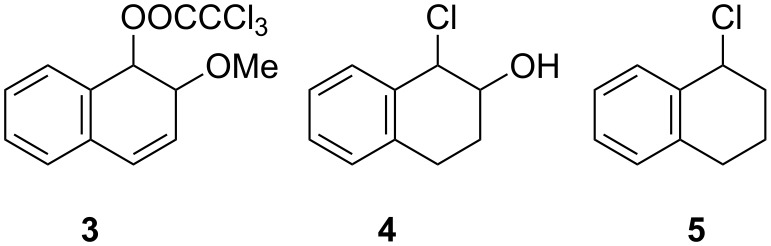
Substrates for solvolysis measurements.

The purpose of the present work was to determine whether the same differentials apply to solvolysis reactions of dihydrodiol derivatives for which one of the hydroxy groups has been converted to a more reactive leaving group. In the case of benzene dihydrodiol it is hard to functionalise the hydroxy group without triggering aromatisation to phenol, at least in the case of the *cis*-diol. The same is true of the *cis*-1,2-dihydrodiol of naphthalene. However, prior methylation of the 2-hydroxy group of this substrate led to the preparation of *cis*- and *trans*-1-trichloroacetoxy-2-methoxy-1,2-dihydronaphthalenes (**3**) which were stable enough to be isolated and purified (Figure 2). This allowed us to compare a ratio of rate constants for the solvolyses of these two isomers with the corresponding ratio for solvolyses of *cis*-and *trans*-1-chloro-2-hydroxy-1,2,3,4-tetrahydronaphthalene (1-chloro-2-tetralol, **4**), which are similar in structure but lack a 3,4-double bond and yield carbocations which cannot undergo deprotonation to form aromatic products.

Finally, to allow the influence of the β-hydroxy group on the rate of solvolysis to be assessed, a rate constant for solvolysis of the corresponding tetrahydronaphthalene substrate lacking a β-hydroxy group, namely 1-chloro-1,2,3,4-tetrahydronaphthalene (**5**), has been measured.

## Results

### *Cis*- and *trans*-1-trichloroacetoxy-2-methoxy-1,2-dihydro-naphthalenes (3)

Rate constants for solvolyses of *cis*- and *trans*-1-trichloroacetoxy-2-methoxy-1,2-dihydronaphthalene (**3**) were measured spectrophotometrically in aqueous acetonitrile and were recorded for different solvent compositions (Table 1). Addition of the acetonitrile to water solubilised the substrates which are precipitated in pure water. A rate constant in water for the *cis*-substrate was extrapolated from measurements at different solvent compositions plotted as log *k* against *Y*_OTs_ (Supporting Information File 1, Figure S1) [4–5]. For the *trans* isomer the solvolysis was too slow for measurements over a wide range of solvent compositions and rate constants could conveniently be measured only for 10 and 20% (v/v) acetonitrile. A rate constant in water was crudely extrapolated from the ratio of rate constants for the *cis* and *trans* isomers at these two solvent compositions. Values in water are shown in brackets in Table 1.

**Table 1:** Rate constants for solvolysis of *cis*- and *trans*-1-trichloroacetoxy-2-methoxy-1,2-dihydronaphthalene (**3**) in acetonitrile-water mixtures at 25 °C.

% MeCN	10^3 ^*k*_obs_(s^−1^) *cis*	10^3^* k*_obs_(s^−1^) *trans*	% solvolysis^a^ *trans*	*Y*_OTs_^b^	*k_cis_*/*k_trans_*

50	0.63			1.4	
40	0.84			1.8	
30	2.31			2.3	
20	6.75	0.0053*^c^*	33	3.2	1.28 × 10^3^
10	20.1	0.0122^c^	16	3.7	1.65 × 10^3^
0	(35)	(0.019)^c^		3.9	(~1.8 × 10^3^)

^a^Measured by HPLC. ^b^Values interpolated from measurements recorded for solvent mixtures made up by weight from [5] ^c^Rate constant for solvolysis corrected for competing hydrolysis.

**Scheme 2:**
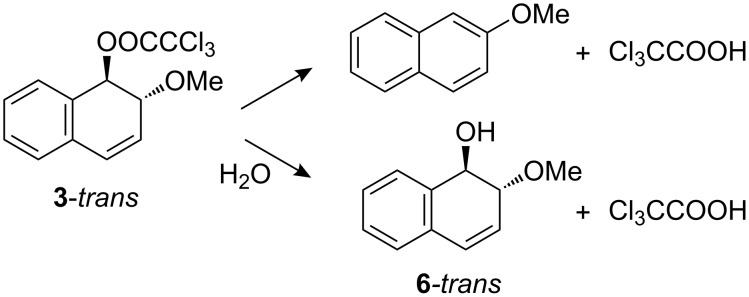
Products of solvolysis and (ester) hydrolysis of *trans*-1-trichloroacetoxy-2-methoxy-1,2-dihydronaphthalene.

In the case of the more slowly reacting *trans* isomer there was a competing hydrolysis of the trichloroacetate ester group to give *trans*-2-methoxy-1,2-dihydro-1-naphthol (**6**-*trans*), as shown in Scheme 2. The fraction of hydrolysis was determined by HPLC and was found to increase with increasing water content and amounted to 67% in 10% acetonitrile.

Surprisingly, 10% of the hydrolysis product was also identified in the products from solvolysis of the *cis*-trichloroacetate (**3**-*cis*). This was unexpected because the solvolysis of this substrate is more than one thousand times faster than that of its *trans* isomer whereas the rate of hydrolysis should remain practically unchanged. In so far as the hydrolysis product was not present in the reactants and the fraction of hydrolysis was independent of solvent composition, the most likely explanation would seem to be that the hydrolysis is catalysed by the *cis*-methoxy group.

The supplementary data record salt effects for the solvolysis of the *cis*-trichloroacetate ester (**3**-*cis*). For sodium acetate, the rate in 20% acetonitrile increased by a factor of close to two at a concentration of 0.4 M, whilst smaller effects were observed for both sodium perchlorate and sodium azide. A little surprisingly, sodium trichloroacetate showed a small negative salt effect leading approximately to a halving of the rate at a salt concentration of 1 M. This is unlikely to be a common ion effect, because loss of a β-proton from a naphthalenium ion intermediate to form the aromatic product (naphthol) is expected to be too fast [6–7]. This conclusion is confirmed by the lack of saturation of the effect and the normal salt effects observed for sodium acetate and sodium azide, which would be expected to trap a carbocation intermediate more effectively than the trichloroacetate anion. The normal salt effect exerted by the sodium azide also confirms that the solvolysis proceeds via the formation of a carbocation intermediate rather than by an S_N_2 mechanism.

### 1-Chloro- and 1-chloro-2-hydroxy-1,2-tetrahydronaphthalenes

**Scheme 3:**
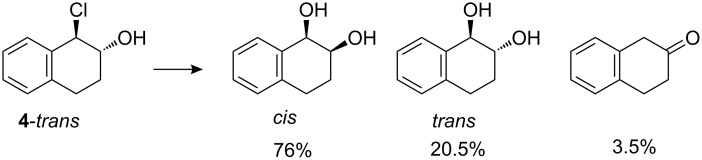
Products of solvolysis of *trans*-1-chloro-2-hydroxy-1,2,3,4-tetrahydronaphthalene.

Rate constants for solvolysis of *cis*- and *trans*-1-chloro-2-hydroxy-1,2,3,4-tetrahydronaphthalenes (**4**) were measured in aqueous solution by monitoring small but easily measurable changes in the UV absorbance. Values obtained were 8.1 × 10^−3^ s^−1^ and 1.6 × 10^−2^ s^−1^ for the *cis* and *trans* isomers, respectively. Product analyses showed the formation of practically the same ratio of *cis* to *trans* diols from *cis* and *trans* reactants together with a small amount of 2-tetralone. The product distribution for the *trans* isomer is shown in Scheme 3. The corresponding figures for the *cis* isomer are 76% *cis* and 20% *trans* dihydrodiols plus 4% 2-tetralone.

The similarity of the product ratios for *cis* and *trans* isomers argues for a common carbocation intermediate in the solvolysis reactions. The possibility of significant reaction via an epoxide intermediate is made unlikely by the predominant formation *cis*-dihydrodiol from the *trans*-chlorohydrin reactant and the formation of similar products from both isomers. Evidence of partial reaction via an epoxide intermediate in buffers at higher pH, which could favour epoxide formation, will be reported elsewhere [8].

**Figure 3:**
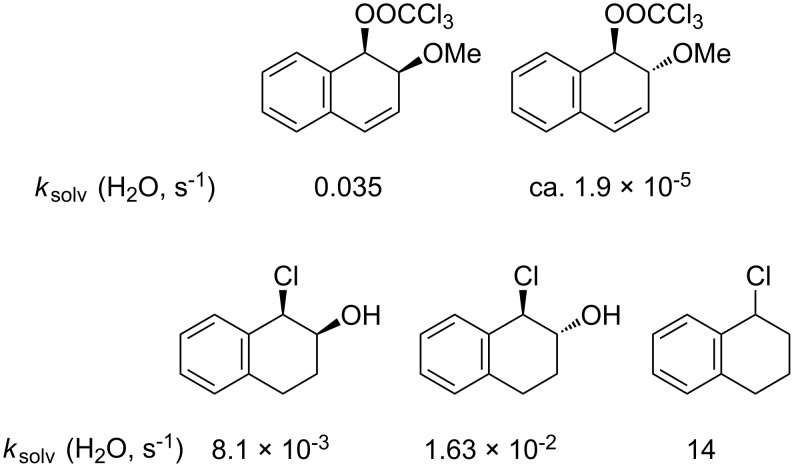
Rate constants for aqueous solvolyses.

For the 1-chlorotetrahydronaphthalene (**5**), solvolysis in water could not be measured directly, but a value of 14 s^−1^ was obtained by extrapolation of a plot of log *k* against *Y*_OTs_ for aqueous acetonitrile mixtures (Supporting Information File 1, Figure S1). The measured and extrapolated rate constants in water for the five substrates studied are summarised under the appropriate structures in Figure 3.

## Discussion

**Figure 4:**
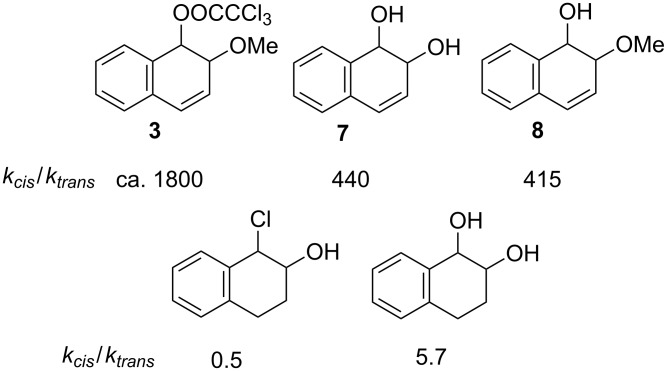
*Cis*/*trans* reactivity ratios for β-hydroxycarbocation forming reactions.

The major conclusion to be drawn from the measurements described above is that solvolysis of *cis* and *trans* 1-trichloroacetoxy-2-methoxy-1,2-dihydronaphthalenes (**3**) show an even larger advantage for reaction of the *cis* isomer, with *k_cis_*/*k_trans_* = ~1800, than the acid-catalysed reaction of the corresponding dihydrodiols **7** (*k_cis_*/*k_trans_* = 440) or the similar 2-methoxy-1,2-dihydronaphthols **8** (*k_cis_*/*k_trans_* = 415) [8]. Moreover, this advantage is lost for the chlorohydrins of the non-aromatic double bond of 3,4-dihydronaphthalene (as it is for the corresponding dihydrodiol). These measurements are summarised in Figure 4.

It is clear that the β-OH and β-OMe substituents exert a profound influence on the relative reactivity of *cis*- and *trans*-dihydrodiol isomers and their derivatives functionalised at the 1-position when the dihydrodiol is derived from an aromatic double bond. A question that arises is: does this selectivity derive from an unusually reactive *cis* isomer or an unusually unreactive *trans* isomer? Evidence on this point comes from examining the effect of the β-hydroxy group on the rate of reactions of the *cis* and *trans* isomers.

Comparison of rate constants for the aqueous solvolysis of *cis*- and *trans*-1-chloro-2-hydroxy-1,2-dihydronaphthalenes (**4**) with the rate constant for the corresponding substrate lacking a hydroxy group (**5**) in Figure 3 shows that the hydroxy slows the rate by a factor of 860 in the case of the *trans* isomer and 1700 in the case of the *cis*. These rate retardations seem a little large when compared with early estimates of the effect of a β-hydroxy or methoxy group in solvolysis reactions, which gave *k*_H_/*k*_OH_ ~100 [9–11]. However, for β-oxy substituents it is difficult to separate a rate-retarding (inductive) effect from a competing acceleration arising from participation of oxygen as a neighbouring group [12–13].

As shown in Table 2, more recent estimates of the rate-retarding effect have been based on incorporating oxygen into a structural framework which prevents its participation as a neighbouring group. An example of this constraint is provided by comparison of solvolyses of the *exo*- and *endo*-norbornyl brosylate with an oxygen atom at the 7-position (**9**) with the corresponding substrate lacking an oxygen. This leads to a rate retardation of 2000- fold for the *exo*-isomers and 6000 fold for the *endo* [14] which is comparable to the differences we observed and, indeed, is similar to the effect of oxygen reported for the monocyclic tetrahydrofuranyl ring of **10** which solvolyses 1030 times more slowly than cyclopentyl brosylate [15].

**Table 2:** Solvolysis reactions: oxygen substituent effects on reactivity^a^.

	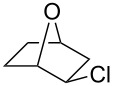	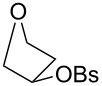	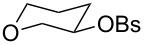	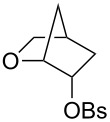
	**9**	**10**	**11**	**12**

k_CH2_/k_O_	2000	1030	18	80000
Temp	85 °C	85 °C	85 °C	25 °C
Solvent	20% aq EtOH	AcOH	AcOH	AcOH

^a^data from [14–16].

These effects are much larger than that of oxygen in the 3-position of the tetrahydropyranyl brosylate (**11**), which slows the rate only by a factor of 18. However, in this case, participation of oxygen as a neighbouring group is well established [15]. Participation by oxygen in a six-membered ring is excluded for solvolysis of *endo* (although not *exo*) norbornyl brosylate substituted with oxygen at the 4-position (**12**). Comparison with the carbocyclic substrate in this case shows a rate retardation of 80,000 [16]. This value has been considered exceptionally large and an additional rate-retarding effect has been attributed to ring strain induced by the presence of oxygen in the six-membered ring of the bicyclic carbocation. Surprisingly, while the solvolysis of *trans*-2-methoxycyclohexyl tosylate has been well studied [17], there appear to have been no measurements reported for its *cis* isomer.

**Figure 5:**
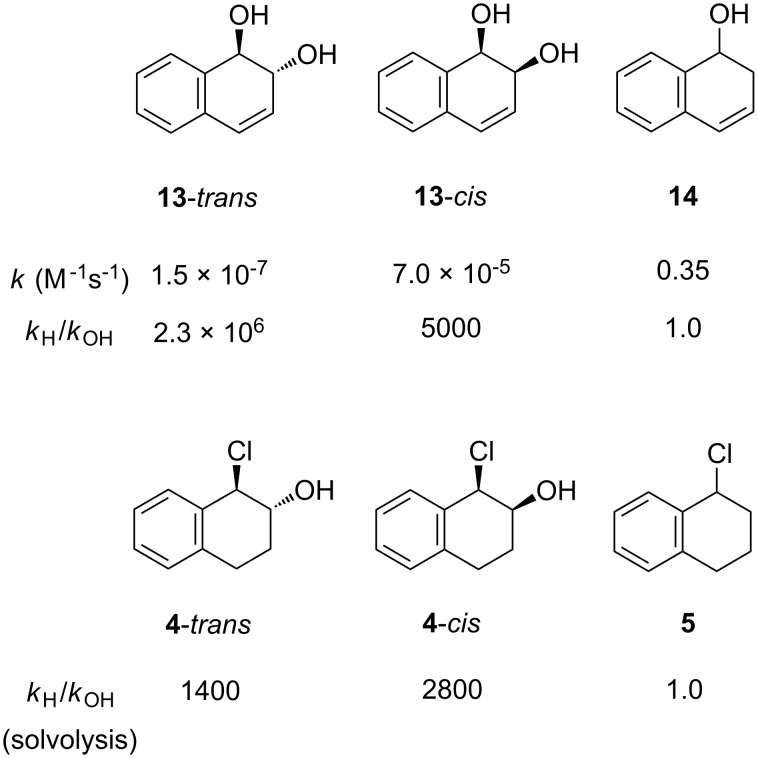
Comparison of the effect of a β-hydroxy group on the reactivity of *cis* and *trans* di- and tetrahdronaphthalene substrates.

We cannot compare these rate ratios directly with those for the solvolyses of *cis*- and *trans-*1-trichloroacetoxy-2-methoxydihydronaphthalenes (**3**) because, as noted in the introduction, the parent 1-trichloroacetoxy-1,2-dihydronaphthalene is too reactive to allow isolation and kinetic measurements. However, rate constants have been measured for the acid-catalysed reactions of *cis* and *trans*-naphthalene 1,2-dihydrodiols **13**-*cis* and **13**-*trans* (reacting at the 1-position) and may be compared with the corresponding alcohol lacking a β-hydroxy substituent, namely the 1-hydroxy-1,2-dihydronaphthalene (**14**) [3], as shown in Figure 5.

It can be seen from this figure that a β-hydroxy substituent has similar effects on the reactivity of *cis*-dihydro and tetrahydro substrates, with a rate retarding effect of 5000 for **13**-*cis* in Figure 5, compared with 2800 for solvolysis of the *cis* β-hydroxychlorotetrahydronaphthalene **4**. In contrast, the effect is much greater in the dihydro case for the *trans*-substituent, i.e., 2.3 × 10^6^ for **13**-*trans* compared with 1400 for solvolysis ot **4**-*trans*. Measurements for benzene and phenanthrene dihydrodiols confirm that, whereas the ratio of *cis*-rate constants to those of the corresponding alcohols, which lack a β-hydroxy group, remains roughly constant, those for the *trans*-dihydrodiols increase sharply as the aromaticity of the double bond increases [18].

**Scheme 4:**
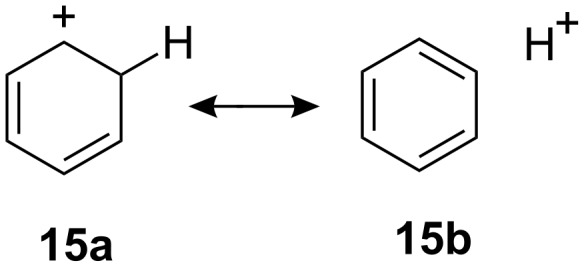
‘Aromatic’ hyperconjugation for the benzenium ion.

These comparisons suggest that that the rate of reaction of the *cis*-dihydrodiol is ‘normal’ while that of its *trans* isomer is abnormally slow. This is consistent with the interpretation already given for the difference in reactivities between the *cis* and *trans* isomers **3**, namely that the arenium ion intermediates are strongly stabilised by hyperconjugation between the carbocationic charge centre and a β-C–H bond, and that this effect is amplified by the contribution of an aromatic structure to the no-bond resonance form (**15b**) of the valence bond representation of this interaction **15a**↔**15b** in Scheme 4 [3].

**Scheme 5:**
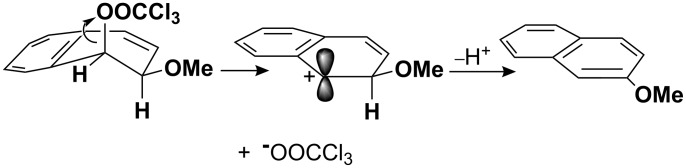
Stereochemistry of carbocation formation from solvolysis of *cis*-1-trichloroacetoxy-2-hydroxy-1,2-dihydronaphthalene.

The interaction operates effectively for arenium ion intermediates formed from alcohols (arene hydrates) and *cis*-dihydrodiols or their derivatives which, as illustrated in Scheme 5, can be formed in a conformation in which an axial C–H bond bond is positioned for hyperconjugation with the positive charge. This is a consequence of a stereochemical constraint requiring that the leaving group departs from an axial position to facilitate delocalisation of the carbocationic charge. However, for the *trans*-dihydrodiols, or the *trans*-1-trichloroacetoxy-2-methoxy-1,2-dihydronaphthol **3**-*trans*, the same constraint forces the β-OH group into an axial position. Although delocalisation of the charge appears to be highly effective when this position is occupied by a β-hydrogen atom (C–H bond), the interaction becomes very much less favourable when occupied by a β-OH group (C–OH bond).

The work describing these favourable and unfavourable hyperconjugative interactions has hitherto been based largely on carbocation formation involving an H_2_O leaving group characteristic of acid-catalysed dehydration of alcohols and *cis* and *trans* dihydrodiols of aromatic and aliphatic carbon–carbon double bonds (**3**). The purpose of the present study was to determine whether the same dependence on stereochemistry of a β-hydroxy group would be observed for solvolytic reactions. Our conclusion, that it is, is based on a comparison of measurements of absolute and relative reactivities of *cis* and *trans* isomers in conventional solvolytic reactions with chloride ion as a leaving group (yielding a carbocation not corresponding to a protonated aromatic molecule) with the solvolytic formation of naphthalenium ions in which the leaving group is a trichloroacetate anion. The only noteworthy difference between the two classes of reactions (i.e., dehydrations and solvolyses) is a slightly larger difference in reactivity between *cis* and *trans* methoxy groups for the solvolytically generated arenium ions (2000 compared with 415) which perhaps represents a more product-like transition for the poorer trichloroacetate than the H_2_O leaving group. In sum, the influence of an enhanced hyperconjugation identified in the reactions of arene dihydrodiols is fully corroborated by the present study of β-hydroxy carbocation-forming solvolysis reactions.

## Experimental

Purchased reagents were generally used without purification. *Cis*-naphthalene dihydrodiol (*cis*-1,2-dihydroxy-1,2-dihydronaphthalene) was prepared by oxidative fermentation of naphthalene [19] and was kindly provided by D. R. Boyd of the Queen’s University of Belfast. Naphthalene oxide and *trans*-1-hydroxy-2-methoxy-1,2-dihydronaphthalene (**6**-*trans*) were prepared as described by Jerina and co-workers [20]. *Trans*-1,2-dihydroxy-1,2-dihydronaphthalene was prepared by the method of Platt and Oesch [21]. *Cis*- and *trans-*1,2-dihydroxy-1,2,3,4-tetrahydronaphthalene [22–24], the corresponding *trans*-1-chloro-2-hydroxy-1,2,3,4-tetrahydronaphthalene [25–26] and 1-chloro-1,2,3,4-tetrahydronaphthalene [25] were also prepared by literature methods.

***cis*-1-Hydroxy-2-methoxy-1,2-dihydronaphthalene (6-*cis*).** To a solution of *cis*-1,2-dihydroxy-1,2-dihydronaphthalene (0.5 g, 3.1 mmol) in DMF (20 mL), was added sodium hydride as a 60% dispersion on mineral oil (0.15 g, 6.2 mmol) followed over 10 min by dimethyl sulfate (0.77 g, 6.18 mmol). The mixture was stirred at room temperature for 20 h, quenched with 1 mL of acetic acid and diluted with water (50 mL). It was then extracted with diethyl ether (2 × 50 mL) and the combined ether layers washed with water (2 × 50 mL) and dried over sodium sulfate. The solvent was removed under reduced pressure to yield a residue consisting of two regioisomeric monomethylated products and the dimethylated product (*cis*-1,2-dimethoxy-1,2-dihydronaphthalene). Purification by chromatography allowed separation of the dimethylated product but the monomethylated products were obtained as a three to one mixture of the desired product with its regioisomer (0.08 g, 15%) (*R_f_* 0.65, 20% ethyl acetate in pentane). NMR data for the principal isomer and analytical data for the mixture were as follows.

^1^H NMR (CDCl_3_) δ 2.6 (bs, 1H, OH), 3.45 (s, 3H), 4.0 (t, *J* = 4.4 Hz, 1H), 4.78 (bs, 1H), 6.08 (dd, *J* = 9.6, 3.9 Hz, 1H), 6.59 (dd, *J* = 9.6 Hz, 1H), 7.1–7.54 (m, 4H); *m/z* GC-MS) 156 (M-H_2_O); (Found C 74.3, H 7.0; C_11_H_12_O_2_ requires C 75.0, H 6.9).

***cis*-1-Trichloracetoxy-2-methoxy-1,2-dihydronaphthalene (3-*cis*).** To a 3:1 mixture (0.1 g, 0.56 mmol) of *cis*-1-hydroxy-2-methoxy-1,2-dihydronaphthalene (**6**-*cis*) and its regioisomer (see above) in dichloromethane (10 mL), were added pyridine (45 mg, 0.57 mmol), DMAP (7 mg 0.05 mmol) and trichloroacetic anhydride (0.21 g, 0.68 mmol) and the solution was stirred at room temperature for 2 h. Evaporation of the solvent gave a crude product mixture from which the desired product was separated by flash chromatography (*R_f_* 0.61, 10% ethyl acetate in pentane) to give a light yellow oil (0.08g, 73%). ^1^H NMR (CDCl_3_) δ 3.4 (s, 3H), 4.31 (m, 1H), 6.05 (dd, *J* = 9.91, 2.39 Hz, 1H), 6.11 (d, *J* = 4.35 Hz, 1H), 6.58 (dd, *J* = 9.81, 1.81 Hz, 1H) 7.15–7.31 (m, 4H).

***trans*-1-Trichloracetoxy-2-methoxy-1,2-dihydronaphthalene (3-*trans*).** To a solution of *trans*-1-hydroxy-2-methoxy-1,2-dihydronaphthalene (**6**-*trans)* (0.1 g, 0.56 mmol) in dichloromethane (10 mL), pyridine (45mg, 0.57 mmol) and DMAP (7 mg, 0.05 mmol) were added. The mixture was stirred for 5 min and trichloroacetic anhydride (0.21g, 0.68 mmol) was added over 5 min followed by stirring for 2 h at room temperature. Evaporation of the solvent gave a crude product which was purified by preparative TLC (5% ether in pentane) to yield the desired product as a light yellow-coloured liquid (0.14 g 77%); (*R_f_* 0.73 10% ether in hexane); ^1^H NMR (CDCl_3_) δ 3.4 (s, 3H), 4.29 (ddd, *J* = 7.53, 3.24, 1.48 Hz, 1H), 6.09 (dd, *J* = 9.87, 3.32 Hz, 1H), 6.28 (d, *J* = 7.55 Hz, 1H), 6.59 (dd, *J* = 9.89, 1.00 Hz, 1H) 7.15–7.31 (m, 4H); *m*/*z* (GC-MS) 158.1 (321.5 –Cl_3_CCOOH).

***cis*-1-Chloro-2-hydroxy-1,2,3,4-tetrahydronaphthalene.** A solution containing 0.05 mL of 4 M HCl in anhydrous dioxane and 0.2 mL of anhydrous THF was added dropwise to a solution of tetrahydronaphthalene-1,2-oxide (100 mg, 0.68 mmol) in anhydrous THF (2 mL) and the mixture allowed to stand at room temperature for 10 min. The solvent and excess HCl were then removed under reduced pressure to yield a crude product which was purified by TLC (10% ether in pentane) to give a colourless liquid (40 mg, 32%); ^1^H NMR (CDCl_3_) δ 1.96 (m, 1H), 2.09 (m, 2H), 2.91 (m, 2H), 3.1 (m, 1H), 4.12 (m, 2H), 5.33 (d, *J* = 3.0 Hz, 1H), 7.11–7.37 (m, 4H); ^13^C NMR (CDCl_3_) 26.56, 27.82, 65.55, 69.14,126.44, 128.85, 128.87, 130.71, 134.56, 135.57; *m*/*z* (GC-MS) 182.5.

**Kinetic measurements and product ratios.** In general, rate constants for solvolysis reactions were measured by injecting 20–25 μL of a ~0.01 M solution of substrate in acetonitrile into 2 mL of water or aqueous acetonitrile in a cuvette and monitoring the reaction from the change in the UV spectrum. For 1-chloro-1,2,3,4-tetrahydronaphthalene rate constants (s**^−^**^1^) were measured for the following acetonitrile water solvent mixtures: 50% MeCN, 0.0320; 60% MeCN, 0.0106; 70% MeCN, 0.00335; 80% MeCN, 0.00082. A plot of log *k* versus *Y*_OTs_ and extrapolation gave a rate constant of ~2.3 s**^−^**^1^ for pure water.

For both the trichloroacetate esters of 2-methoxy-1,2-dihydro-1-naphthol and the *cis*-and *trans*-chlorohydrins of 1,2-dihydronaphthalene product analyses were carried out by HPLC. In a typical procedure 25 μL of ~0.01M solution of the substrate in acetonitrile were injected into 150 μL of water or aqueous acetonitrile mixture. After allowing time for completion of the reaction, the products were analysed by reverse phase HPLC on a C18 column with a flow rate of 0.25 mL/min and detection at 270 nm. For the trichoroacetic esters, the product proportions were required to partition measured rate constants between contributions from pathways for solvolysis and hydrolysis.

## Supporting Information

File 1 Experimental part.
