# Supplementary material for: β-Hydroxy carbocation intermediates in solvolyses of di- and tetra-hydronaphthalene substrates
Source: Beilstein J Org Chem. 2010 Nov 3;6:1035–42. doi: 10.3762/bjoc.6.118 (PMC2981814; doi:10.3762/bjoc.6.118)
Supplement: File 1 — Experimental part. [file Beilstein_J_Org_Chem-06-1035-s001.pdf]

Supporting Information

for

β-Hydroxy carbocation intermediates in solvolyses of di- and tetra-hydronaphthalene substrates

Jaya S. Kudavalli and Rory A. More O'Ferrall*

Address: School of Chemistry and Chemical Biology, University College Dublin, Belfield, Dublin 4, Ireland

Email: Rory A. More O'Ferrall* - rmof@ucd.ie

Jaya S Kudavalli - kjsnarayana@yahoo.co.in

* Corresponding author

This Supporting Information contains two items: The first is plots of log *k* versus Y_{OTs} for the solvolysis of substrates considered in the main manuscript to allow extrapolation of measurements in aqueous acetonitrile mixtures to pure water. The second is a table of rate constants arising from measurements of salt effects on the solvolysis reactions.

The only solvent parameter measured for acetonitrile water mixtures is Y_{OTs} [1,2]. This parameter was used to correlate kinetic measurements, therefore even though the leaving groups studied were chloride or trichloroacetate rather than tosylate. Indeed Y_{OTs} has only been measured in the range 0–60% acetonitrile. We extended this range by studying the solvolysis of 1-chloro-2,3-cyclohexene in both methanol

water mixtures and acetonitrile water mixtures. The measurements in aqueous methanol were correlated by the relationship log k = −1.34 + 1.123 Y_{OTs}, From measurements for the same substrate in aqueous acetonitrile, values of Y_{OTs} were inferred from the relationship Y_{OTs} = (log k + 1.34)/1.123 [3]. The derived values (−0.14, 80% MeCN; 0.44, 70% MeCN; 0.87, 60% MeCN; 1.40, 50% MeCN; 1.81, 40% MeCN; 2.21, 30% MeCN) were deemed satisfactory for extrapolating a value of log k for 1-chloro-1,2,3,4-terahydronaphthalene in pure water. Plots of log k versus Y_{OTs} for this substrate (filled circles) and for *trans*-1-trichloroacetoxy-1,2-dihdyronaphthalene (open circles) are shown in Figure S1. For the latter substrate directly measured values of Y_{OTs} were used, although values appropriate to solvents mixed by volume had to be interpolated from measurements recorded for solvent mixtures made up by weight. The equations for the two correlations are log k = −2.93 + 1.045Y_{OTs} for the 1-chlorotetrahydronaphthalene and log k = −4 + 0.65Y_{OTs} for the *trans*-1-trichloroacetoxy-1,2-dihdyronaphthalene. Rate constants in water were interpolated by taking Y_{OTs} for water as 3.9.

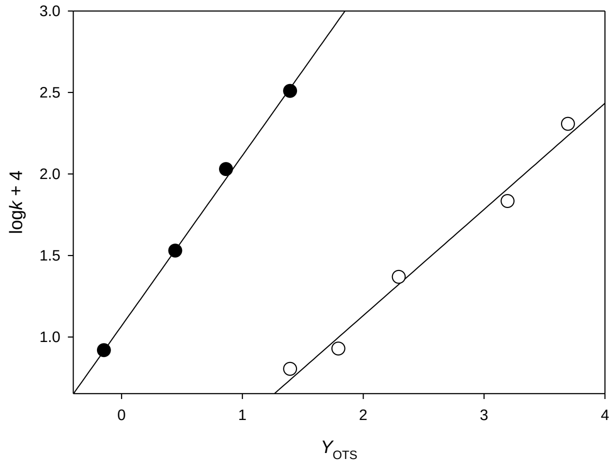

Figure S1: Plots of log k versus Y_{OTs} for solvolysis of 1-chloro-1,2,3,4-terahydronaphthalene (filled circles) and *trans*-1-trichloroacetoxy-1,2-dihdyro-naphthalene (**10**-*trans*, open circles) in acetonitrile water mixtures at 25 °C.

Salt effects on the solvolysis of *cis*-1-trichoracetoxy-2-hydroxy-1,2-dihydronaphthalene. Table S1 lists measurements of rate constants for solvolysis in 80:20 (v:v) aqueous acetonitrile in the presence of varying concentrations of sodium perchlorate, sodium acetate, sodium azide and sodium trichloroacetate in the concentration range 0–0.8 M. The rate constant in the absence of added salt is 6.75×10^{-3} s^{-1}.

Table S1: First order rate constant (s^{-1}) for the solvolysis of *cis*-1-trichoracetoxy-2-hydroxy-1,2-dihydronaphthalene at different concentrations of added salts in 80% aqueous acetonitrile at 25 °C.

Concn	10^3k_{obs}	10^3k_{obs}	10^3k_{obs}	10^3k_{obs}
M	$NaClO_4$	NaN_3	NaOAc	$NaOOCCCl_3$
0.2	7.39	7.61	7.94	5.85
0.4	7.90	8.21	10.7	4.75
0.6	8.10			4.12
0.8	8.44			

References

1. Bentley, T. W.; Llewellyn, G. *Prog. Phys. Org. Chem*. **1990,** *17,* 121–158. doi:10.1002/9780470171967.ch5
2. Bunton, C. A.; Mhala, M. M.; Moffatt, J. R. *J. Org. Chem.* **1984,** *49,* 3637–3639. doi: 10.1021/jo00193a036
3. Boyd, D. R.; Coyne, D.; Keeffe, J. R.; Kudavalli, J. S.; Lawlor, D. A.; MacCormac, C.; More O'Ferrall, R. A.; Rao, S. N.; Sharma, N. D. *Org. Lett.*, in press.
